# The viral arms race: an interview with Harmit Malik

**DOI:** 10.1242/dmm.052591

**Published:** 2025-09-09

**Authors:** Harmit Malik

**Affiliations:** Basic Sciences Division, Fred Hutchinson Cancer Center, Seattle, WA 98109, USA


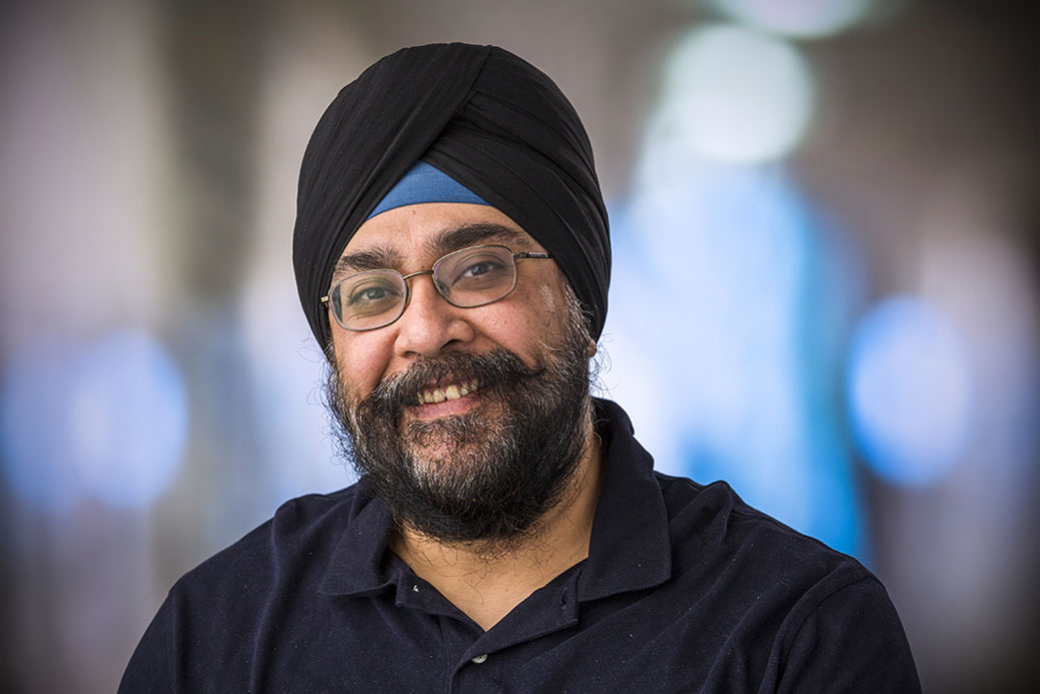


**Harmit Malik.** Photo credit: Fred Hutchinson Cancer Research Center.

Harmit Malik is Professor and Associate Director of the Basic Sciences Division at the Fred Hutchinson Cancer Center in Seattle, WA, USA, where he works on genetic conflict, and the co-evolution of viruses and their hosts. His research spans a range of disease contexts, from present-day viruses and cancer to the ancient viral imprints that past infections have left in our genomes. After initially studying chemical engineering as an undergraduate, Harmit moved from India to the USA for his PhD at the University of Rochester, where he worked on retrotransposon evolution with Dr Thomas Eickbush. He then moved to the Fred Hutchinson Cancer Center to work on the evolution of centromeric histones during his postdoc with Professor Steve Henikoff and stayed there to open his own lab in 2003. Harmit has made significant contributions to our understanding of the ‘molecular arms races’ between viruses trying to evade host immune systems and the host antiviral proteins trying to keep up. In this interview, we discuss the fascinating evolutionary insights hidden in our genomes, his approach to supporting his mentees and the beauty of basic bioscience research.

[…] coming from outside of biology has given me a kind of curiosity that is perhaps its own superpower

**Your undergraduate degree was in chemical engineering. What led you to transition towards biological research?**

Studying chemical engineering, I was surrounded by people who really had a passion for it. Even though I was doing fine in the classes, I realised I didn't have the same dedication. It didn't seem like a good idea to spend your whole life doing something that, perhaps, you were not so passionate about. I had been reading a lot of Richard Dawkins during my anarchist phase of being a teenager. I loved ‘The Selfish Gene’ – it was published decades ago, but it's still super relevant. I ended up taking an ‘introduction to molecular biology’ course and the professor was extremely charismatic and open. He made me realise I could consider a career in biological research. At the time, I was more interested in applied biotechnology but, as I got to learn more about evolution and the basic mechanisms of molecular biology, I found myself reading much more about it than I needed to for my course. I realised this was clearly something I was very curious about, and it would be a mistake not to at least consider it as a career. So, I applied to PhD programmes in the USA and in India. I didn't get into a single PhD programme in India because I only had one biology class on my transcript, but schools in the USA were more used to the idea that people could come into molecular biology from very different backgrounds – physicists, mathematicians … and chemical engineers, as it turned out. The first year of graduate school was quite brutal because there were a lot of undergraduate classes in biology and biochemistry that I had not taken before. Even now, when I've been a faculty member for more than two decades, somebody can tell me something quite fundamental regarding cell biology that I didn't know. But I'm not actually daunted by that because I think coming from outside of biology has given me a kind of curiosity that is perhaps its own superpower – I am generally curious about things that don't make as much sense to me from an outside perspective, and often that's the trigger for new projects in the lab.

**Your lab works on evolutionary ‘arms races’ between viruses and hosts. What has been your most exciting discovery in this area?**

The finding that I think we are best known for still gives me a lot of excitement – maybe I'll even use the word ‘pride’. Molecular biologists tend to focus on what is really conserved in evolution as a proxy for what is functionally important. And that is certainly true – you look at the catalytic residues of an enzyme, and you know that it cannot tolerate any mutations in those residues because that would completely disrupt the catalytic mechanism. In arms races driven by binding affinity, the recognition of a viral protein by an antiviral protein dictates the outcome of infection. These arms races are often driven by rapidly evolving interfaces: the viruses constantly evolve to evade binding, and the immune proteins constantly evolve to restore binding. But what my group was amongst the first to realise was that, instead of focusing on the most-conserved parts of the protein, we began focusing on the least-conserved parts that most people probably considered not that important. These regions are in previously uncharacterized loops, and there was no evidence of evolutionary constraint. But we realised that there was, in fact, evidence of evolutionary constraint: it was the opposite constraint. Instead of conservation, it was about constant innovation; and we realised that we could use this lack of conservation as a proxy to identify precise residues that would dictate the outcomes of viral infections. So, I believe this was the breakthrough discovery to jump start the way we think about host–virus interactions and host–virus evolutionary arms races by focusing on the most rapidly evolving parts of both proteins.

Overall, we have about four times more dead viruses in our genome than all of the protein-coding exons in the genome

**What can be learned by studying the viral remnants of past infection in the genome?**

My background was in transposable elements – these elements in the genome are the ultimate selfish genes really, because they duplicate themselves and their daughter copies make new homes in different parts of the genome. But every time an element jumps, there's a real risk that it might land in a part of the genome that disrupts a core function, destroying the host organism. A lot of principles relating to transposable elements also apply to viruses that integrate into the genome and leave their imprints, like retroviruses do. They leave an accumulated history of insertions spanning years of evolution. It's astonishing to consider that we have more than 100,000 different imprints of ancient viruses sitting in our genomes. Some are very easily recognised as viruses, some are more difficult to recognise, and we need to use approaches – like machine learning – to really delve into this. Overall, we have about four times more dead viruses in our genome than all of the protein-coding exons in the genome – the sheer baggage these viruses encode is astonishing. But the longer these viral remnants reside in the genome, the higher the chance they acquire a useful property. One of the best examples is in the evolution of placental genes in mammals, likely to be due to a happenstance insertion and domestication of a retrovirus that has, ultimately, facilitated live birth. This process occurred multiple times in the evolution of mammalian lineages. So, you begin to realise that each of these imprints – even if we would initially consider them noise – have the potential to evolve into some important function. We become more and more addicted, in a way, to these viral imprints that land in the genome.

**How does understanding viral evolution help our understanding of cancer?**

Cancer predisposition genes pose an evolutionary paradox – they hurt the survival of the species, they hurt fertility so should be selected against. There's a hand-wavy argument that cancer is fundamentally a disease of aging and, so, isn't subject to the same selection pressures because you're past reproductive age by the time it manifests. But, actually, that's not true for many cancers. Many manifest as paediatric cancers. Other cancers, like breast cancer, manifest in prime reproductive years. So, we began to consider the possibility that this is actually an evolutionary echo of a different type of adaptation, where these susceptibility alleles arose and thrived in populations because they were the best solutions to combat viral infections. We think of them as alleles that have lost function and are clearly associated with disease, but they are evolutionary winners in a certain sense, because these are the alleles that survived and thrived in the face of past infections. We and others have begun to make these connections for important genes, including the BRCA family, to understand their participation in viral infections. What we have not done yet is really show that the disease susceptibility alleles are the ones that provided benefit – some of that requires catching them in the act with the right pathogen. But what we can clearly see is that many of these cancer predisposition genes bear the same signatures we see for antiviral proteins and immune proteins, so there's clearly been recurrent selection driven by viruses and pathogens. Exactly why these alleles lead to this trade-off between housekeeping functions that, when compromised, lead to cancer, versus protective functions against pathogens is something we'd like to study more of in the future.

We cannot simply rely on the idea that the therapies currently in clinical trials are going to be enough […] there needs to be a constant influx of new ideas to stay ahead of the arms race

**How important do you feel discovery research and ‘basic’ science is for understanding disease?**

Well, I have a little bit of a biased view on the topic, since I'm a basic scientist myself. The lab has made more and more discoveries with very strong therapeutic implications, and often people ask me why we are not pursuing these further ourselves. Part of it is that I think about this very much as an ecosystem. People have different skills – I have colleagues who are very good at the application side of things and I have other colleagues, including people in my lab, who are very good at the basic science. There are a lot of very smart people at every stage in the ecosystem and, sometimes, we have to acknowledge that we can't all be experts in every step. A lot of basic science discoveries will end up having profound implications in the clinic – if you don't have the full imagination about how to get it there, that's okay, because you're still a very important piece of the jigsaw puzzle and other people can help. If the basic science discoveries didn't exist, then it's quite possible that the well would run dry. We cannot simply rely on the idea that the therapies currently in clinical trials are going to be enough because we already know that – for diseases, such as cancer, and with rapidly evolving viruses – there needs to be a constant influx of new ideas to stay ahead of the arms race. I'd also make a plug for the fact that, ultimately, we are all interested in human disease, but disease research in humans is not ethical or possible. This is why creating and studying model organisms in a high-throughput, low-investment context is incredibly important. We cannot just say ‘okay, we're going to stop work on anything that is not related to human research’, because – actually – it's all relevant to humans.

**Do you think basic science is particularly threatened by cuts to funding?**

Science itself is quite uncertain. We do experiments wondering if they will even work. It's discovery, and you don't know where it's going to lead. It could lead to a billion-dollar company, something like mRNA vaccines or CRISPR-Cas9 gene editing, or it could simply be something that interests you. Sometimes it might appear esoteric from the outside, but there are very smart people dedicated to this work. We shouldn't lose sight of the fact that most of this work is paid for by taxpayers, but funding uncertainty creates a very unstable foundation. If the foundations are weak, people are going to get much more conservative about the science that they're doing and worry that ‘blue-skies research’ is not worth pursuing because it won't get funded. And that would be a mistake because all innovation in science really originates from blue-skies, basic research. The second thing that uncertainty does is send a message to our young trainees – who are our future – that this is not a career option that will provide professional and personal stability. I worry that this kind of uncertainty will mean we lose an entire generation of people, and that would be a loss we might not be able to overcome.

I give a lot of credit to the various trainees, postdocs and students who have come to the lab. I can always suggest an idea but, ultimately, they're the ones who are actually taking the risk.

**You use a range of model organisms in your work, from *Drosophila* to primates. How do you choose a model system to address a specific research question?**

We often start with focusing on what the best question is to study. Once we have done that, we move into the more practical aspects of what the best model organism would be. Bringing a completely new model organism to the lab is a very daunting exercise. There are people who have done that very successfully multiple times, and I give them a lot of credit. We are not those people! However, we have been buoyed by the successes of colleagues who have brought these systems into their labs and recognise that there is real value in collaborating with them, and learning from them. Often there are cases where the perfect model organism provides a huge leg up in terms of relevance to the condition or process that we really want to study. For example, we have studied chromosome segregation – how chromosomes get separated during cell division – for practically the entire time I've been in science, primarily using *Drosophila* and cell lines from humans. But there are some chromosomes in which the entire segregation machinery resides not just in single domains but along the entire chromosome. We call these holocentric chromosomes. Neither humans nor *Drosophila* have holocentric chromosomes, so we needed to find a system that allowed us to study them without having to spend a decade trying to get that organism into the lab. Using literature searches and conversations with colleagues, we arrived on butterflies and moths, so started working on silk moths. Some of this also requires the imagination of the person who's leading the work in the lab. I give a lot of credit to the various trainees, postdocs and students who have come to the lab. I can always suggest an idea but, ultimately, they're the ones who are actually taking the risk. I'm really happy that, in many cases, this has really paid off for them, and their careers have been established based on this work.

**What do you think are the most important things to consider when mentoring junior scientists?**

I think it's really important to know what they want to get out of their training stint in your lab. Some people really want to collect experience with skill sets that the lab is already well-known for. Some want to establish a research programme that they can actually take to their own independent labs. So, very clear communication and making sure our goals are aligned is really important. These relationships can start running into trouble if mistrust creeps in and you're worried about, say, what projects you can take with you and what will stay in the lab. The way we've tried to solve that problem is by agreeing that postdocs will take away absolutely everything that they work on. Often, they'll take away the model organism system and they'll take away the question in its entirety so we do not have any competition with them for at least the first six years of their independent lab. I realise that junior faculty members can be in a really vulnerable position, so I want them to be able to take risks and try something new while they have the relative safety net of a postdoc fellowship, and a well-resourced lab. We are constantly losing projects as postdocs leave the lab but this is a really good outcome for us. Our former postdocs end up doing well, and that can be good advertisement for the lab, but it also forces us to constantly think about what the next big questions are. It also makes the lab itself a very vibrant environment because we have multiple trainees, all going after their own projects without feeling that they're competing with each other.

**With the lab pursuing a range of different projects, what's a key question you'd like to address in the next few years?**

The really cool thing is that my postdocs' or students' latest obsessions often become my latest obsessions. Even knowing full well that this project might leave my lab, it's still really fun to think about. One of our latest obsessions in the lab is investigating the compromises viruses have to make when jumping back and forth between different species, e.g. mosquitoes to humans and back to mosquitoes. They have to deal with completely different temperatures and immune systems, so there must be some compromise. Some of this has already been tackled, and we know that these viruses are compromising aspects of their replication strategies to make sure that they will survive well enough in both hosts, but we really want to understand the molecular nature of that compromise. New technology has allowed us to revisit these very well-established problems and gain new insights. We're discovering all kinds of unusual temperature-sensing strategies in these RNA viruses. I'm super excited about this. It's science fiction for now, but the idea would be to see if we could make a variant of the virus that is so well suited to a mosquito vector that it would outcompete the version of the virus capable of jumping to humans, essentially ‘trapping’ the virus in mosquito hosts.

Our other recent obsession is a bit of an extension of work we've done in the past. We've previously looked backwards at the sequences of host and viral genes, trying to decipher the interaction interfaces; but now we're trying to use combinatorial mutagenesis to go forward and uncover completely new antiviral specificities in existing antiviral genes that might be dormant. Natural selection has not reached these solutions yet but we can reach them quite quickly in the lab, and this has some therapeutic implications, for example, if we could generate antiviral proteins that are really effective against human-adapted flu. There are some ideas where the instantaneous application is obvious, like this, but other ideas will take time and require the right circumstances to have impact. I believe that ideas are good – some ideas can be heretical, but that doesn't mean they're not at least worth considering.

**What do you enjoy doing outside of the lab?**

This is perhaps too revealing a question – I really like reading murder mysteries. When I was growing up in India, I read a lot of P. G. Wodehouse and then grew into Agatha Christie. I think I have read every Agatha Christie mystery novel that has ever been written. But now my latest books to read are these Scandinavian mysteries, often involving serial killers. It's a little bit escapist in a way, to freeze your brain into thinking about something else.

